# Proxy reporting of health-related quality of life for people with dementia: a psychometric solution

**DOI:** 10.1186/s12955-020-01396-y

**Published:** 2020-05-24

**Authors:** S. C. Smith, A. A. J. Hendriks, S. J. Cano, N. Black

**Affiliations:** 1 Department of Health Services Research & Policy, London School of Hygiene & Tropical Medicine, WC1H 9SH, London, UK; 2 Modus Outcomes, Spirella Building, Letchworth Garden City, SG6 4ET UK

**Keywords:** DEMQOL, HRQL, Psychometric, Rasch, Dementia

## Abstract

**Background:**

The growing move towards personalised health and social care systems means that every effort needs to be made to generate patient-reported outcome data. However, the deteriorating nature of dementia can make it difficult for people with dementia to complete self-reported questionnaires and it is often necessary to rely on a family member (proxy) to report on their behalf. There is little evidence to guide how the difference between self- and proxy-reports of health reported quality of life (HRQL) in dementia can be interpreted.

**Methods:**

We recruited people with dementia and their family carers from 78 memory Assessment Services in the UK. We used Rasch measurement methods to investigate whether a HRQL questionnaire known as DEMQOL (self-reported by the person with dementia) and DEMQOL-Proxy (proxy-reported by a family carer) can be placed on the same continuum and whether a revised scoring algorithm, based on this equated model, can be developed that takes account of the relationship between self- and proxy-reports.

**Results:**

In a sample of 1434 patients and 1030 carers, our findings supported equating DEMQOL/DEMQOL-Proxy (overall fit to the model; no mis-fitting items) after addressing specific issues (eight disordered items requiring re-scoring, four pairs locally dependent items, and five items showing DIF). Cross walk tables have been produced.

**Conclusions:**

We have established for the first time that DEMQOL and DEMQOL-Proxy can be placed on the same continuum and that patients and carer proxies are reporting on the same construct when they complete these questionnaires. Where possible both DEMQOL and DEMQOL-Proxy should still be administered together, using the improved scoring algorithm reported here. Where only DEMQOL-Proxy is available, the cross walk tables provide an estimate of DEMQOL for a particular person from their DEMQOL-Proxy score.

## Background

The growing move towards personalised health and social care systems [1, 2] means that every effort needs to be made to generate patient-reported outcome data. People with mild or moderate dementia often have capacity to express their views and preferences. However, the deteriorating cognitive ability experienced by people with more severe dementia often means that it is necessary to rely on a proxy report. Two widely used dementia-specific HRQL questionnaires (QOLAD [3]) and DEMQOL [4, 5] have both self- and proxy-reported versions. However, proxy-reports of HRQL are not substitutable for self-reports from people with dementia [6–8].

Agreement between self- and proxy-reports in dementia is estimated to be moderate at best for both disease-specific [3, 4, 9] and generic patient-reported outcome measures (PROs) [10]. Consistent with wider evidence from other conditions [11], proxy reports of HRQL in dementia tend to be lower than the patients’ own self-reports [5] and there are recognised differences in the predictors of self- and proxy-reported HRQL in dementia [12]. Qualitative work [4] also suggests that there are differences in the type (as well as extent) of issues that are revealed by self- and proxy-reports. For example, patients tend to compare themselves with their peers whereas carers make comparisons with how the patient used to be in the past. Carers also find it difficult to separate the potential impact on their own HRQL. Recommendations have therefore been to use both self- and proxy-reports of HRQL where possible [4].

Yet there are some circumstances where proxy reports may be the only practical way of obtaining information about HRQL for someone with dementia [13]. In research, longitudinal follow-up has sometimes necessitated using only a proxy-report so that the same PRO can be compared across time. In large scale routine data collection (and also some clinical situations), the time and cost of interviewer administering a self-reported questionnaire may be prohibitive and it may not always be feasible to interviewer administer a questionnaire to ask the person with dementia themselves. Understanding of HRQL for people with dementia is therefore limited, either by contradictory scores from both patients and carers, or by relying solely on a proxy-report in the knowledge that this is likely to be different from what the person with dementia themselves would have said. There is little evidence to guide how this difference can be interpreted consistently.

Methodological investigations of this issue have focused on the extent of agreement between self- and proxy-reports and statistical explanations for the extent of proxy-related bias. It is generally agreed that proxy bias will be increased for questionnaire measures with lower internal consistency [14], where the distribution is skewed or where the number of patient/proxy pairs is low [11]. An alternative explanation is that patients and carers are not reporting on the same construct – that is they have a different perception and understanding of what constitutes HRQL. In this case, it would be unlikely that self-and proxy- reports are highly correlated and substituting a carer’s proxy-report for a self-report would be misleading.

Existing HRQL instruments for people with dementia have typically been developed using psychometric methods based on Classical Test Theory [15, 16]. Modern psychometric methods, such as those based on Rasch Measurement Theory [17, 18], provide a way of investigating whether self- and proxy-reports of HRQL are reflecting the same construct of HRQL. Good measurement requires that all the items in a questionnaire can be placed on a single continuum (or “ruler”). Entering both self-reported and proxy-reported items from the same questionnaire into the same Rasch analysis determines the extent to which both sets of items are part of the same continuum (or ruler) of HRQL. If they are, then the proxy-reports can be anchored by the self-reports for the common items. In this way, a revised scoring algorithm can be developed, based on the Rasch model, that calibrates the proxy response by taking account of the equivalent self-reported response. The analysis also generates a cross-walk table between the proxy-reported scores and the estimated equivalent self-reported scores. In practical terms this provides a meaningful way of interpreting proxy-reports of HRQL in dementia. It enables the person with dementia to remain central in discussions of their HRQL even when it is necessary to rely on the proxy-report of a family carer. In other conditions this technique has been used successfully to create a common metric (scale) from multiple instruments measuring mobility in multiple sclerosis [19], but, to our knowledge, this method has not yet been used to equate self- and proxy-reported scores in dementia. We have already demonstrated that Rasch measurement methods improve the scoring for both DEMQOL and DEMQOL-Proxy separately [20]. This paper investigates whether DEMQOL and DEMQOL-Proxy can be placed on the same continuum and consequently whether a revised scoring algorithm, based on this equated model can be developed that takes account of the relationship between self- and proxy-reports.

## Methods

### Sample

The data were collected as part of a large cohort study investigating the effectiveness and cost effectiveness of memory Assessment Centres (MAS) or memory clinics in England. The sample was recruited from the population of people with suspected dementia attending MAS for a first appointment. 78 clinics recruited up to 25 consecutive patients and their accompanying carer (if they had one) between September 2014 and April 2015. People with insufficient English language to understand the consent process or the questionnaire were excluded.

### Data collection - DEMQOL and DEMQOL-proxy

DEMQOL and DEMQOL-Proxy [5] are designed to measure HRQL of people with dementia. DEMQOL is self-reported but interviewer-administered and consists of 28 items. DEMQOL-Proxy has 31 items and although originally developed to be interviewer-administered can also be self-administered. In this study carers were therefore given the DEMQOL-Proxy questionnaire and asked to complete it independently. DEMQOL-Proxy asks carers to give the answer that they think the person with dementia themselves would have given. Both instruments are reported on a 4-point Likert type scale (a lot/quite a bit/a little/not at all). Both DEMQOL and DEMQOL-Proxy are scored so that a higher score indicates better HRQL.

### Psychometric methods

We undertook a Rasch analysis of the DEMQOL and DEMQOL-Proxy items in two phases to determine whether the self- and proxy-reported items could be placed on the same continuum. In phase 1, we stacked the data set (i.e. a vertical arrangement with cases appearing twice, once as self-ratings and once as proxy ratings). We entered all 44 items (59 items, of which 15 are common to the two instruments) into a single Rasch preliminary model. Our previous Rasch analysis of DEMQOL and DEMQOL-Proxy as separate scales showed that the positive emotion items do not form a unidimensional scale with the rest of the items for either DEMQOL or DEMQOL-Proxy and recommended removing these items from the model [20]. Three of these items are common to DEMQOL and DEMQOL-Proxy, 2 were unique to DEMQOL and 2 were unique to DEMQOL-Proxy. We therefore removed these 7 items from the dataset before beginning the analysis reported here. After rescoring 8 items with disordered thresholds (also known to be disordered in previous Rasch analyses of DEMQOL and DEMQOL-Proxy separately [20]), we anchored the model by the DEMQOL responses to the remaining 12 common items.

**Fig. 1:**
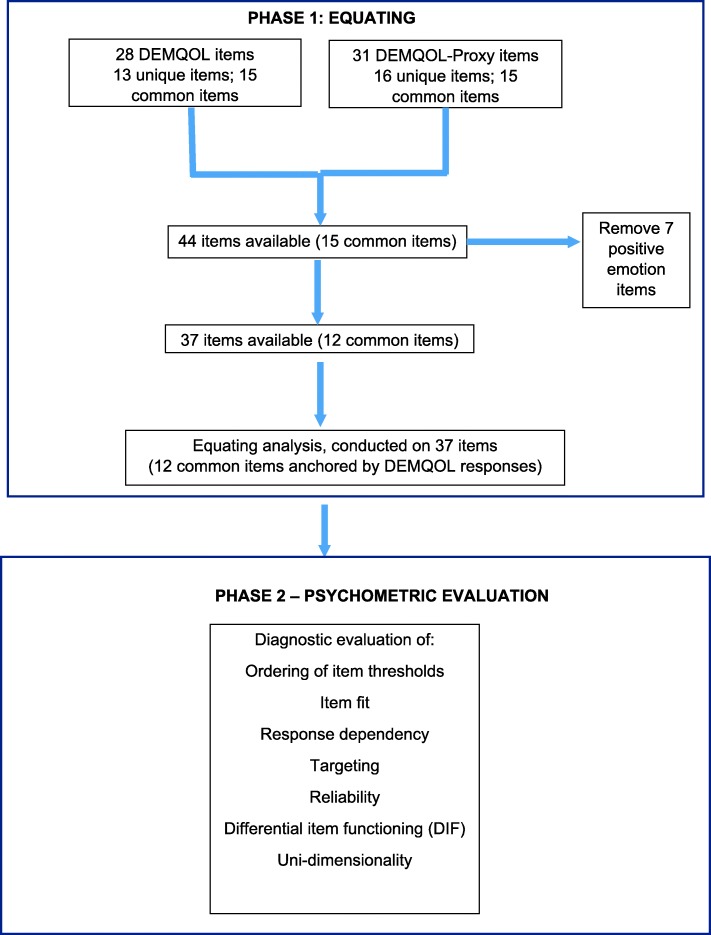
Overview of the two methods phases

In phase 2 with this anchored model with 37 items, we evaluated overall fit to the Rasch model and used a series of diagnostic tests to identify anomalies in the data that indicated aspects of the scale that were not working as intended. To do this, we evaluated the extent to which the following properties were true: each item fit the model; the thresholds between each of the response options were ordered; different groups within the data (assuming the same amount of the construct being measured) did not show differences in scores; items were independent of each other; items in the instrument represented a uni-dimensional construct; items were reliable and targeted at a similar range of the construct being measured as exists in the people being measured. The criteria that were applied are well established in the psychometric literature (see for example [21–23]) Each of these diagnostic criteria is described in more detail below. From the final model, we extracted the Rasch scores for both DEMQOL and DEMQOL-Proxy to form the cross-walk table. All analyses were conducted using RUMM 2030 software [24], using the unrestricted Rasch model for ordered responses. Because the Chi-square statistic is highly sensitive to sample size, all tests of significance were performed in an adjusted sample size of *N* = 500. Bonferroni corrections were also applied. The overview of the two phases is summarised in Fig. 1.

#### Ordering of item thresholds

We evaluated the structure of the response categories to determine whether response options were being used as intended. The threshold map gives a visual display of the thresholds between each category (i.e. the point at which each response option becomes the most likely response). The thresholds should be ordered in the same way as the response options. Disordered thresholds can indicate where respondents have misunderstood or been unable to use response categories consistently. More optimal response option structures can be explored post-hoc by collapsing or re-scoring the disordered thresholds.

#### Item fit

We evaluated both the overall fit of the data to the Rasch model using Chi square and also the fit of each item to the Rasch model. Individual item fit was evaluated both statistically (fit residuals within the range of +/− 2.5 and non-significant Chi square) and graphically (visual inspection of the item characteristic curves, illustrating the extent of agreement between observed and expected scores for groups of people with similar HRQL).

#### Response dependency

After taking account of the “Rasch” factor we evaluated the extent to which the residuals were related. Pairs of items where the residuals were correlated > 0.3 were considered for re-calibration.

#### Targeting

A robust measurement instrument should show good targeting. The spread of item (threshold) locations along the scale should be similar to the spread of person locations in the sample. That is, there is a match between the range of HRQL measured by the DEMQOL/DEMQOL-Proxy items and the range of HRQL in the sample. We evaluated the targeting of the equated DEMQOL/DEMQOL-Proxy scale by visually inspecting the targeting diagram.

#### Reliability

Reliability was evaluated using the Person Separation Index (PSI; similar to Cronbach’s alpha). A value > 0.7 is considered adequate.

#### Differential item functioning (DIF)

DIF occurs when particular groups (e.g. men or women, people with mild or severe cognitive impairment) respond differently to the same item, even though they have the same level of HRQL. This is evaluated using ANOVA to assess whether the construct being measured is different in the groups across the different levels of the construct being measured (class intervals). In this analysis groups were defined as person with dementia (PWD) sex, PWD age group (quartiles), and disease severity (≥ 24 versus < 24 MMSE or equivalent based on published cut offs indicating dementia). For DEMQOL-Proxy we additionally defined groups according to the sex and age group (quartiles) of the carer and relationship to the PWD (spouse, son/daughter, other) and whether the questionnaire was reported by PWD or the carer. We evaluated uniform DIF looking for a significant main effect for the particular group and non-uniform DIF by looking for a significant interaction between the and the class intervals. If uniform DIF is found, the item can be re calibrated as a separate item for each level of the particular group where DIF was found. Items showing non-uniform DIF may need to be investigated and/or removed from the item set.

#### Uni-dimensionality

Application of the Rasch model requires unidimensional data, which means that any subset of items measuring the same construct should yield highly similar person location estimates. Our previous work has investigated the uni-dimensionality of DEMQOL and DEMQOL-Proxy as separate scales. In this paper we aimed to determine whether and to what extent items from DEMQOL and DEMQOL-Proxy formed a uni-dimensional scale. To this end, two subsets of items were created, the first consisting of the 23 DEMQOL items and the second consisting of 26 DEMQOL-Proxy items. We used a series of independent *t*-tests to investigate whether < 5% of the estimates for these two subsets differed significantly (percentage of individual *t*-tests outside the range ± 1.96). We computed Wilson 95% confidence intervals [25].

#### Equated scores for DEMQOL and DEMQOL-proxy

We used the final equated model without resolving items showing DIF and/or local dependency to derive Rasch based scores, as our previous work [20] had demonstrated a strong, positive relationship (ICC ≥ 0.97) between Rasch based scores resolving these items and the scores derived from the same model without resolving. In this analysis, Rasch based scores were extracted for both DEMQOL and DEMQOL-Proxy based on the final equated model and a cross-walk table produced. This enables a user to “cross walk” from a DEMQOL-Proxy score back to the equivalent DEMQOL score.

## Results

### Sample characteristics

A total of 1434 patients and 1030 carers were available to the analysis. Table 1 shows the characteristics of the sample. The sample has been shown elsewhere to be representative of people with dementia attending Memory Assessment Services and of those recruited about half were subsequently diagnosed with dementia [26].

**Table 1:** Sample characteristics

	Age range (mean; SD)	Gender %	Ethnicity %	Cognitive function (%)*	Type of carer %
**People with dementia**	42–98 (77.9; 8.5)	52 female 48 male	94.5 White British 1.6 Black British 0.9 Asian British 1.1 Mixed 1.8 Other	MMSE < 24 = 702 (58.7) MMSE ≥24 = 494 (41.3) Missing = 238	N/A
**Carers**	16–94 (65.9; 13.6)	69 female 31 male	95.2 White British 1.6 Black British 0.9 Asian British 1.6 Mixed 0.7 Other	N/A	61 spouse/partner 32 son/daughter (in law) 7 other

* Where MMSE score not available, ACE-III (≤ 82 vs. > 82), ACE-R (≤ 82 vs. > 82), MOCA (< 22 vs. ≥ 22), M-ACE (≤ 21 vs. > 21), KOLT (≤ 22 vs. > 22), or TYM (≤ 42 vs. > 42) score used based on established cut-offs for screening

### Ordering of item thresholds

After re-scoring the 8 disordered items, no other items were disordered.

### Overall fit to the model and item fit

Overall the items fit the Rasch model (*p* = 1.0). 15 items had fit residuals >+/− 2.5 (see Table 2). Of these, two DEMQOL items (*p*-* worried about getting on with people close to you*, *p*- worried about getting help when needed) and one DEMQOL-Proxy item (*c - worried about using money to pay for things)* also showed item characteristic curves that indicated that these items may be over-discriminating. No items showed significant Chi square statistics.

**Table 2:** Item fit for DEMQOL-23 and DEMQOL-Proxy 26 items

Item	Location	SE	FitResid	DF	ChiSq	DF	Prob
p- worried or anxious (common)	0.541	0.026	−2.072	2284.74	1.415	9	0.998
p- Frustrated (common)	0.748	0.025	−3.001	2271.34	8.143	9	0.520
P- Sad (common)	0.264	0.027	−2.393	2270.38	22.499	9	0.007
p- lonely	−0.024	0.037	3.439	1323.75	4.577	9	0.870
p- Distressed (common)	−0.231	0.028	−5.489	2265.60	4.497	9	0.880
p- Irritable (common)	0.190	0.028	−0.654	2275.17	3.006	9	0.964
p- fed up (common)	0.617	0.026	−1.061	2269.43	8.435	9	0.491
p- wanted to do things but couldn’t	1.043	0.032	4.251	1320.88	6.899	9	0.648
p- forgetting things happened recently (common)	0.854	0.026	−1.588	2288.57	17.075	9	0.048
p- forgetting people	0.145	0.035	2.811	1328.54	5.283	9	0.809
P forgetting the day (common)	0.331	0.025	3.742	2289.53	7.56	9	0.579
p- thoughts being muddled (common)	0.305	0.026	−7.016	2281.87	9.364	9	0.404
p- difficulty making decisions (common)	0.072	0.026	−4.921	2287.61	11.869	9	0.221
p- poor concentration	0.487	0.035	−2.185	1323.75	3.52	9	0.940
p- not enough company (common)	−1.211	0.064	−1.069	2281.87	11.387	9	0.250
p- getting on with people close	−0.914	0.084	−2.711	1322.79	8.098	9	0.524
p- getting the affection needed	−1.312	0.096	−1.854	1321.84	4.958	9	0.838
p- people not listening	−0.328	0.041	0.141	1317.05	2.279	9	0.986
p- making self understood (common)	−0.175	0.028	−0.929	2225.40	2.015	9	0.991
p- getting help when needed	−1.114	0.089	−3.693	1318.01	8.816	9	0.454
p- getting to the toilet in time	−0.889	0.083	−1.079	1319.92	1.479	9	0.997
p- how you feel in yourself	0.187	0.037	−4.092	1316.09	7.078	9	0.629
p- your overall health	0.417	0.035	−1.204	1323.75	1.280	9	0.998
c-memory in general	0.639	0.043	−0.236	954.29	1.074	9	0.999
c-forgetting things that happened long ago	−0.421	0.042	2.948	961.95	5.120	9	0.824
c- forgetting names	0.662	0.040	0.444	959.07	1.387	9	0.998
c-forgetting where s/he is	−0.811	0.046	−1.545	955.25	4.321	9	0.889
c-keeping him/herself clean	−1.640	0.100	−0.695	963.86	1.383	9	0.998
c-keeping him/herself looking nice	−1.529	0.097	−0.478	960.99	2.217	9	0.988
c-getting things from the shops	−0.454	0.041	−1.378	950.46	1.497	9	0.997
c-using money to pay	−1.295	0.091	−2.863	951.42	7.185	9	0.618
c-looking after finances	−0.254	0.038	0.496	940.89	3.265	9	0.953
c-things taking longer	0.393	0.040	−4.000	958.12	6.425	9	0.697
c-getting in touch with people	−0.442	0.042	−2.453	951.42	3.275	9	0.952
c-not being able to help others	−0.425	0.041	−0.035	955.25	2.428	9	0.983
c-not playing a useful part	−0.205	0.040	−2.939	956.20	3.651	9	0.933
c-his/her physical health	0.363	0.039	1.180	960.99	2.376	9	0.984

**Key:** Items denoted **p-(item)** are unique to DEMQOL and self-reported by the person with dementia; items denoted **p-(item) (common)** are in both questionnaires, but in this analysis the data from the self-reported DEMQOL was used; items denoted **c (item)** are unique to DEMQOL-Proxy and reported by the carer

**Note** Fit residuals in blue are outside the acceptable range of +/− 2.5. Location = average item threshold location (logit). ChiSq = chi square value (comparing observed with expected values); *p* = chi square probability

### Response dependency

Four pairs of items had residual correlations > 0.3, 1 pair for DEMQOL (*p*-*worried about getting on with people close to you/p-getting the affection that you want* = 0.41) and 3 pairs for DEMQOL-Proxy (*c* - *worried about keeping clean/c - worried about keeping looking nice* = 0.66; *c - worried about using money/c - worried about looking after finances* = 0.40; *c - worried about not being able to help others/c - worried about not playing a useful part* = 0.41).

### Targeting

**Fig. 2:**
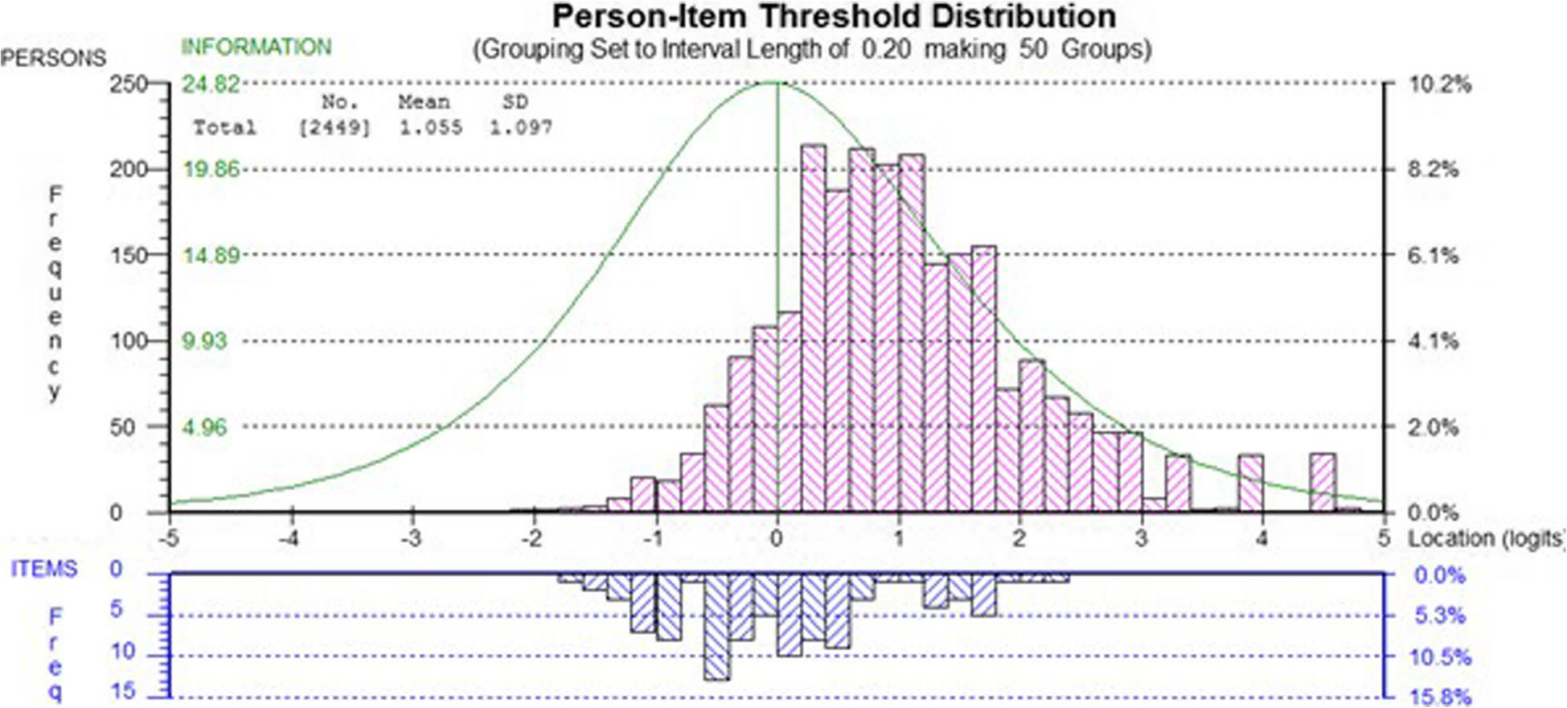
Person item theshold distribution (targeting diagram)

The targeting of item locations to the location of the sample along the scale was adequate but could be improved. The person item threshold distribution (see Fig. 2) suggests that there is a proportion of people who have better HRQL than is represented by the highest HRQL items.

### Reliability

The equated scale showed good reliability (PSI = 0.89).

### Differential item functioning (DIF)

Five items showed significant main effects (uniform DIF); two DEMQOL items (*p-feeling lonely and p-worried about not having enough company*) showed DIF by relationship; three further DEMQOL items (*p-feeling sad, p-worried about forgetting things that happened recently, p-worried about difficulty making decisions*) showed DIF by reporter. No items showed significant interactions between any of the person factors and class intervals (non-uniform DIF).

### Uni-dimensionality

The two sets of measurements differed significantly for 11 cases (0.46%; 95% CI: 0.25%; 0.82%) at the 5% level indicating that the equated scale is uni-dimensional.

### Equated scores for DEMQOL and DEMQOL-proxy

**Fig. 3:**
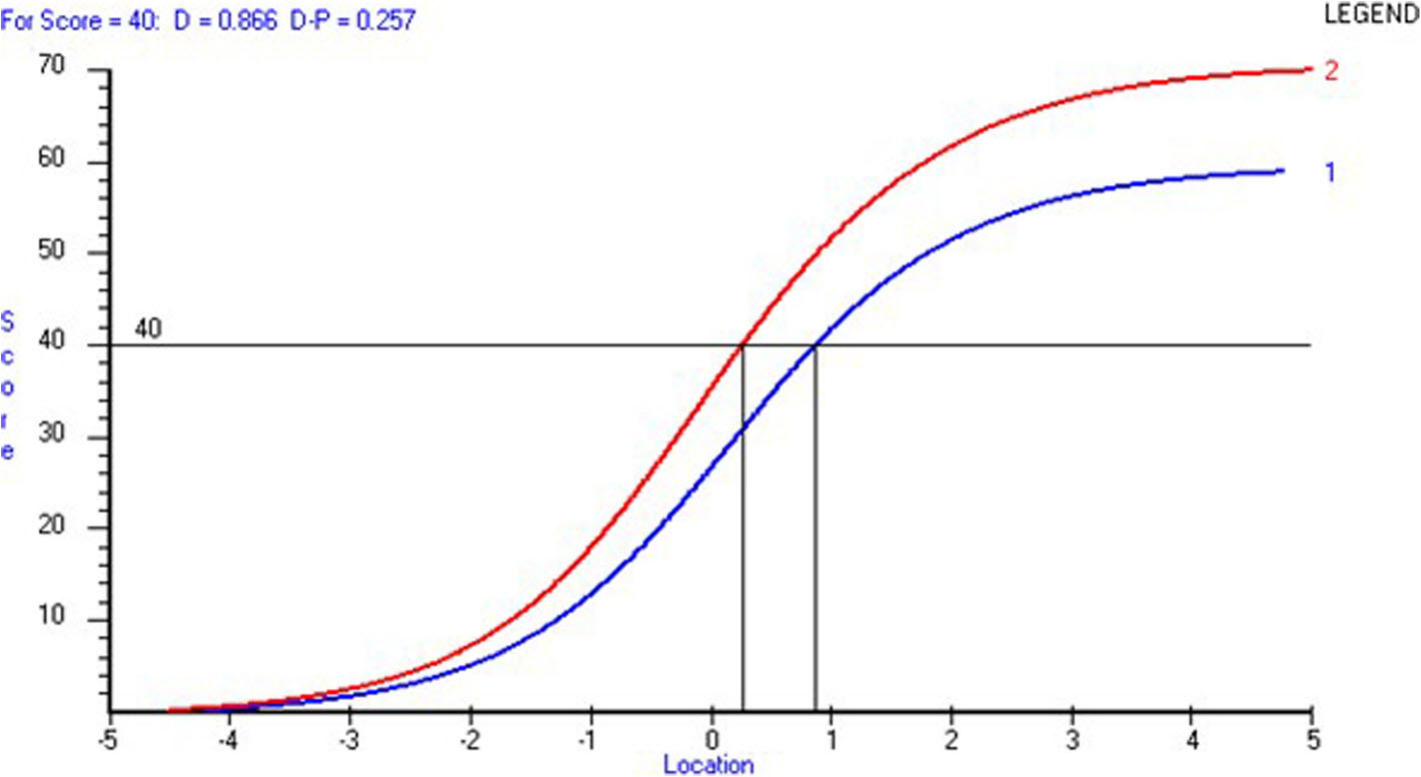
Relationship between DEMQOL-23item score and DEMQOL-Proxy-26 item scores

Figure 3 illustrates the relationship between DEMQOL and DEMQOL-Proxy scores. Taking the example of a patient with a raw score of 40 (on DEMQOL), the equivalent equated Rasch based DEMQOL score in logits (blue line) would be 0.866 logits (this can be transformed to a 0–100 scale, giving a value of 59) and the DEMQOL-Proxy raw score equivalent to a logit value of 0.866 would be 50. For the same raw score on DEMQOL-Proxy (i.e. 40) the equivalent equated Rasch based DEMQOL-Proxy score in logits (red line) would be 0.257 logits (which when transformed to 0–100 scale gives a value of 53). The cross-walk table allows these equivalent scores to be obtained for all points on the scale. Thus, it is possible to obtain an estimate of a DEMQOL score even when data need to be collected via a proxy report.

## Discussion

We have used Rasch measurement theory to establish that DEMQOL and DEMQOL-Proxy can successfully be placed on the same continuum. The results reported here confirm that both patients and carer proxies are reporting on the same construct when they complete these questionnaires. Therefore, we have developed an improved scoring algorithm that links DEMQOL and DEMQOL-Proxy scores and cross-walk tables to obtain an estimate of DEMQOL for a particular person from their DEMQOL-Proxy score. The cross walk table is provided as a supplementary file to this paper.

The cross walk tables provide a unique solution to the problem of proxy-reporting of HRQL in dementia. For the first time, it is possible to rely on a proxy-report, but also to be able to estimate the equivalent self-report. This has two implications. First, DEMQOL-Proxy scores can be interpreted with confidence because they are anchored by the DEMQOL responses for the common items, thus, keeping the perspective of the person with dementia central even within proxy reports. Second, where it is not possible to obtain a self-report, this can be reliably estimated from the proxy-scores via the cross-walk table. Further, in longitudinal studies where data collection begins with self-report, but at later time points has to rely only on proxy-reports, the cross-walk tables enable the data to be interpreted on the same scale, making meaningful comparison over time possible. Together with the validated self-administered version of DEMQOL-Proxy recently developed [27] we now have the potential to collect HRQL data from people with dementia in large population samples. This was previously limited by the need to rely on expensive, time consuming interviewer-administration of DEMQOL-Proxy and by the knowledge that DEMQOL-Proxy scores were different to DEMQOL but with no established scoring link between them. Obviously, if a person with dementia is able to self-report DEMQOL this is preferable and DEMQOL and DEMQOL-Proxy should continue to be administered together wherever possible.

The equated Rasch scores can also be used at the individual level, providing a robust contribution to clinical decision making. The data meet the requirements of the Rasch model and the resulting scores have the advantage that, unlike scores derived from Classical Test Theory, generate individual standard errors. The scores can therefore be used as one source of information within a clinical encounter to guide discussion or as a way of sharing decision making. The ability to cross-walk from DEMQOL-Proxy to DEMQOL scores means that the person with dementia’s view can remain central to this process even when s/he is no longer able to complete a questionnaire.

The applicability of the scoring algorithms presented here is limited by the relatively mild nature of the samples. Our sample was recruited at first appointment at MAS and a large proportion had a MMSE score ≥ 24. However, this was an appropriate sample for this first test of whether self- and proxy-reports could be equated as we are confident in the self-report of people with mild/moderate dementia [5]. With increasing severity, the insight of people with dementia declines and reliability of self-reports potentially decreases. We do not know whether and to what extent DEMQOL and DEMQOL-Proxy can be equated with samples that include people with more severe dementia. Nor do we know the point of severity at which equating is no longer possible and people with dementia and their carers are no longer reporting the same construct. We are collecting follow up data of this sample and future psychometric work will determine how far along the longitudinal trajectory (6, 12, 24 months) DEMQOL and DEMQOL-proxy can still be equated.

The sample is also limited by the need to administer the questionnaires in the English language as this is how DEMQOL and DEMQOL-Proxy were originally validated. Although there has been a small amount of work to validate the instruments for other languages and cultures this has been limited to European languages and, on the whole, does not enable data collection in diverse ethnic groups where English is not spoken. Proxy reporters may be influenced by a wider range of factors than just their perception of the person with dementia’s experience of HRQL and carers also find it hard to separate their own experience from that of the person with dementia. Culturally-specific models of caring may also influence the nature of proxy-reporting in other ethnic groups. It would be worthwhile to determine the extent to which the construct of HRQL is similar (and can be placed on the same continuum) for people with dementia and their carers amongst other ethnic groups.

This method of scoring DEMQOL/DEMQOL-Proxy does not include the five positive emotion items for reasons that are documented elsewhere [20]. The positive emotion items remain important for HRQL in dementia and DEMQOL/DEMQOL-Proxy should always be administered in its original format (28 items for DEMQOL and 31 items for DEMQOL-Proxy). All the available DEMQOL/DEMQOL-Proxy scores (original scores based on Classical Test Theory [5], DEMQOL-U scores [28] and the Rasch based equated scores reported here) are based on the same conceptual framework, but each uses a slightly different combination of items depending on the intended purpose. The development of each of these scores has selected a sub-set of items using clear criteria appropriate to the purpose for which the score is to be put. Future users should choose the score which best reflects their purpose, but the questionnaire should always be administered in the original paper based format.

Future research that uses the cross walk tables developed here will contribute to a body of evidence about HRQL for people in dementia where the person with dementia’s view is kept central. This will potentially increase the quality of policy and applied health care decisions by increasing the validity of evidence on such decisions is based.

We conclude that DEMQOL/DEMQOL-Proxy should continue to be administered together where possible as they measure complementary aspects of HRQL. The equated Rasch scores for DEMQOL and DEMQOL-Proxy reported here provide more robust scoring algorithms that can be used at both the individual and population levels. When a self-report of DEMQOL is no longer possible from the person with dementia we can now interpret DEMQOL-Proxy scores with confidence because they are anchored to the equivalent DEMQOL responses. Furthermore, it is possible to cross-walk from DEMQOL-Proxy to an estimate of the equivalent DEMQOL score. Together with the previously validated self-administered version of DEMQOL-Proxy [27] this provides a practical and economic method of collecting large scale population data about HRQL in dementia.

## Conclusions

We have established that DEMQOL and DEMQOL-Proxy can be placed on the same continuum and that patients and carer proxies are reporting on the same construct when they complete these questionnaires. Where possible both DEMQOL and DEMQOL-Proxy should still be administered together, using the improved scoring algorithm reported here. Where only DEMQOL-Proxy is available, the cross walk tables provide an estimate of DEMQOL for a particular person from their DEMQOL-Proxy score.

## Supplementary information

**Additional file 1.** Supplementary file with cross walk tables for DEMQOL-Proxy-26 and DEMQOL-23.

## Data Availability

The datasets generated and analysed during the current study are not yet publicly available, but can be requested from the corresponding author.

## Abbreviations

HRQL: Health related quality of life
DIF: Differential item functioning
PRO: Patient reported outcomes
PSI: Person separation index
ICC: Intra class correlation
PWD: Person with dementia
