# Supplementary material for: Proxy reporting of health-related quality of life for people with dementia: a psychometric solution
Source: Health Qual Life Outcomes. 2020 May 24;18:148. doi: 10.1186/s12955-020-01396-y (PMC7245851; doi:10.1186/s12955-020-01396-y)
Supplement: Supplementary file 1 — Additional file 1. Supplementary file with cross walk tables for DEMQOL-Proxy-26 and DEMQOL-23. [file 12955_2020_1396_MOESM1_ESM.docx]

**SUPPLEMENTARY FILE WITH CROSS WALK TABLES
FOR DEMQOL-PROXY-26 AND DEMQOL-23**

**INTRODUCTION**

The cross walk table below refers to 4 scores. These are defined as follows:

**ORIGINAL DEMQOL-PROXY SCORE (26 ITEMS).** This is the raw score for DEMQOL-Proxy reported in the accompanying paper.

**ORIGINAL DEMQOL SCORE (23 ITEMS).** This is the raw score for DEMQOL-Proxy reported in the accompanying paper.

As these two raw scores were based on the items that fit the Rasch model, they are slightly different to the original scores (previously published see Smith et al 2005). To use the cross walk table in your own data you will need to derive these raw scores (for DEMQOL-23 and DEMQOL-Proxy-26) using syntax that is available on request ([sarah.smith@lshtm.ac.uk](mailto:sarah.smith@lshtm.ac.uk)). To simply use the original scores for DEMQOL (28 items) and DEMQOL-Proxy (31 items) you should continue to use the already available coding syntax (available from <https://www.bsms.ac.uk/research/neuroscience/cds/research/demqol.aspx>).

**RASCH DEMQOL-PROXY SCORE (DP-26).** This is the Rasch score for DEMQOL-Proxy reported in the accompanying paper, transformed to 0-100 for ease of use and rounded up to the nearest integer.

**RASCH DEMQOL SCORE (D-23).** This is the Rasch score for DEMQOL reported in the accompanying paper, also transformed to 0-100 and rounded up to the nearest integer.

These are the equivalent Rasch scores for DEMQOL-Proxy and DEMQOL that should be reported as transformed Rasch scores for DEMQOL-Proxy-26 and DEMQOL-23 respectively.

Further SPSS code to automate the generation of equivalent scores via the cross walk within a particular dataset is available on request from the authors ([sarah.smith@lshtm.ac.uk](mailto:sarah.smith@lshtm.ac.uk)).

Please note, it is not meaningful to compare the original DEMQOL-Proxy score (far left column) directly with the original DEMQOL score (far right column).

**HOW TO USE THE CROSS WALK TABLE:**

1. Locate the original DEMQOL-PROXY-26 score in the far left-hand column.
2. Look across to the next column to locate the equivalent Rasch DEMQOL-Proxy (DP-26) score
3. Look across to the third column to locate the equivalent Rasch DEMQOL score (D-23).

**EXAMPLE**: an original DEMQOL-Proxy-26 score of 15 (see box 1 below) has an equivalent transformed Rasch score of 37 (see box 2 below). The cross walk to the transformed Rasch DEMQOL score (D-23) shows that for a proxy-reported transformed Rasch score of 37, the equivalent patient-reported transformed Rasch score is 41 (see box 3 below).

**DEMQOL-PROXY-26/DEMQOL-23 CROSS WALK TABLE FOR RASCH MODEL BASED SCORES**

| ORIGINAL  DEMQOL-PROXY SCORE  (26 ITEMS) | **RASCH DEMQOL-PROXY SCORE  (DP-26)** | **RASCH DEMQOL SCORE  (D-23)** | ORIGINAL  DEMQOL SCORE (23 ITEMS) |  |
| --- | --- | --- | --- | --- |
| 0 | **0** | **0** | 0 |  |
| 1 | **11** | **13** | 1 |  |
| 2 | **16** | **19** | 2 |  |
| 3 | **20** | **23** | 3 |  |
| 4 | **23** | **25** | 4 |  |
| 5  **Box 1**  Original DEMQOL-Proxy score of 15  **Box 2**  Equivalent Rasch score for an original score of 15  **Box 3** Cross walked Rasch DEMQOL score | **25** | **28** | 5 |  |
| 6 | **27** | **30** | 6 |  |
| 7 | **29** | **32** | 7 |  |
| 8 | **30** | **33** | 8 |  |
| 9 | **31** | **35** | 9 |  |
| 10 | **32** | **36** | 10 |  |
| 11 | **34** | **37** | 11 |  |
| 12 | **35** | **38** | 12 |  |
| 13 | **36** | **39** | 13 |  |
| 14 | **36** | **40** | 14 |  |
| 15 | **37** | **41** | 15 |  |
| 16 | **38** | **42** | 16 |  |
| 17 | **39** | **43** | 17 |  |
| 18 | **40** | **44** | 18 |  |
| 19 | **40** | **45** | 19 |  |
| 20 | **41** | **45** | 20 |  |
| 21 | **42** | **46** | 21 |  |
| 22 | **42** | **47** | 22 |  |
| 23 | **43** | **47** | 23 |  |
| 24 | **43** | **48** | 24 |  |
| 25 | **44** | **49** | 25 |  |
| 26 | **45** | **50** | 26 |  |
| 27 | **45** | **50** | 27 |  |
| 28 | **46** | **51** | 28 | |
| 29 | **46** | **52** | 29 | |
| 30 | **47** | **52** | 30 | |
| 31 | **48** | **53** | 31 | |
| 32 | **48** | **54** | 32 | |
| 33 | **49** | **54** | 33 | |
| 34 | **49** | **55** | 34 | |
| 35 | **50** | **56** | 35 | |
| 36 | **50** | **56** | 36 | |
| 37 | **51** | **57** | 37 | |
| 38 | **51** | **58** | 38 | |

**DEMQOL-PROXY-26/DEMQOL-23 CROSS WALK TABLE CONTINUED**

| ORIGINAL  DEMQOL-PROXY SCORE  (26 ITEMS) | **RASCH DEMQOL-PROXY SCORE  (DP-26)** | **RASCH DEMQOL SCORE  (D-23)** | ORIGINAL  DEMQOL SCORE (23 ITEMS) |
| --- | --- | --- | --- |
| 39 | **52** | **59** | 39 |
| 40 | **53** | **59** | 40 |
| 41 | **53** | **60** | 41 |
| 42 | **54** | **61** | 42 |
| 43 | **55** | **62** | 43 |
| 44 | **55** | **63** | 44 |
| 45 | **56** | **64** | 45 |
| 46 | **56** | **65** | 46 |
| 47 | **57** | **66** | 47 |
| 48 | **58** | **67** | 48 |
| 49 | **58** | **68** | 49 |
| 50 | **59** | **70** | 50 |
| 51 | **60** | **71** | 51 |
| 52 | **61** | **73** | 52 |
| 53 | **62** | **74** | 53 |
| 54 | **62** | **76** | 54 |
| 55 | **63** | **79** | 55 |
| 56 | **64** | **82** | 56 |
| 57 | **65** | **86** | 57 |
| 58 | **66** | **91** | 58 |
| 59 | **67** | **100** | 59 |
| 60 | **69** |  |  |
| 61 | **70** |  |  |
| 62 | **71** |  |  |
| 63 | **73** |  |  |
| 64 | **75** |  |  |
| 65 | **77** |  |  |
| 66 | **79** |  |  |
| 67 | **82** |  |  |
| 68 | **86** |  |  |
| 69 | **92** |  |  |
| 70 | **100.00** |  |  |

**References:**

Smith SC, Lamping DL, Banerjee S, Harwood RH, Foley B, Smith P, et al. Development of a new measure of health‑related quality of life for people with dementia: DEMQOL. Psychological Medicine. 2007; 37,737-46.
